# Complete genome sequence of invertebrate iridovirus IIV22A, a variant of IIV22, isolated originally from a blackfly larva

**DOI:** 10.4056/sigs.5059132

**Published:** 2014-04-20

**Authors:** Benoît Piégu, Sébastien Guizard, Tan Yeping, Corinne Cruaud, Arnault Couloux, Dennis K. Bideshi, Brian A. Federici, Yves Bigot

**Affiliations:** 1 UMR INRA-CNRS 7247, PRC, Centre INRA de Nouzilly, Nouzilly, France; ^2^ Department of Entomology and ^3^Interdepartmental Graduate Programs in Cell, Molecular and Developmental Biology, University of California, Riverside, California, USA; 4 CEA/Institut de Génomique GENOSCOPE, Evry CEDEX, France; 5 California Baptist University, Riverside, California, USA

**Keywords:** iridoviridae, iridovirus, dsDNA virus, insect, invertebrate, MITE, intein

## Abstract

Members of the family Iridoviridae are animal viruses that infect only invertebrates and poikilothermic vertebrates. The invertebrate iridoviruses 22 (IIV22) and 25 (IIV25) were originally isolated from a single sample of blackfly larva (*Simulium* spp., order Diptera) collected from the Ystwyth river near Aberystwyth, Wales. Recently, the genomes of IIV22 (197.7 kbp) and IIV25 (204.8 kbp) were sequenced and reported. Here, we describe the complete genome sequence of IIV22A, a variant that was isolated from the same pool of virions collected from the blackfly larva from which the IIV22 virion genome originated. The IIV22A genome, 196.5 kbp, is smaller than IIV22. Nevertheless, it contains 7 supplementary putative ORFs. Its analysis enables evaluation of the degree of genomic polymorphisms within an IIV isolate. Despite the occurrence of this IIV variant with IIV22 and IIV25 in a single blackfly larva and the features of their DNA polymerase, we found no evidence of lateral genetic transfers between the genomes of these two IIV species.

## Introduction

The Iridoviridae consists of a family of viruses with a large double-stranded DNA (dsDNA) that is encapsidated within an icosahedral capsid. Their genomes have a circularly permuted configuration with terminal redundancy. As a consequence, the map of their genomes is represented as a circular molecule. Each virion encapsidates a single linear DNA molecule, the ends of which are located at different positions on the map of the dsDNA genome [1]. Genome replication includes distinct nuclear and cytoplasmic phases [1]. The family Iridoviridae is currently organized into five genera: Chloriridovirus, Iridovirus, Lymphocystivirus, Megalocytivirus and Ranavirus. Members of the two first genera have a host range restricted to invertebrate species (arachnids, cephalopods, crustaceans, insects, mollusks, nematodes, and polychetes; for review, see [2]), whereas members of the three other infect only poikilothermic vertebrates (fishes, amphibians and reptiles). These viruses are members of the nucleoplasmic large DNA viruses (NLCDV) [3], now referred to as the Megavirale [4].

Among invertebrate iridoviruses (IIVs), the model species for the genus Chloriridovirus is IIV3 [1,5], the only species reported in this genus. The model species for genus Iridovirus is IIV6 [6]. The genome of IIV1 has not been sequenced, and so far only two species, IIV1 and IIV6, are recognized as representatives of the genus Iridovirus in the last report of the International Committee for Virus Taxonomy (ICTV) [1]. Ten other related viruses that may be iridovirus species await biological and genomic data before it can be determined whether they are valid species of this genus. Recently, the genomes of three members of the genus Iridovirus were published: IIV9 [7], IIV22 [8] and IIV25 [9]. These three viruses were found to have more genes in common with IIV3 than with IIV6, but even so their nucleic acid sequences exhibit major differences with that of IIV3. The conservation of their gene organization and the similarity between the nucleic acid sequences, 75 to 85% between IIV22 and IIV9 or IIV25 on 60% of their length and 85 to 92% identical between IIV9 and IIV25 over 88% of their length, indicate that these viruses are more related to each other than to any other IIVs. Aside from their conserved features, the genomes of these three viral species also differ by the presence of inverted regions, inteins, transposons, the number of members of some gene families, the total number of genes, as well as the location of some of these. It was previously proposed to gather virus species in a species complex called Polyiridovirus [10,11].

**Figure 1:**
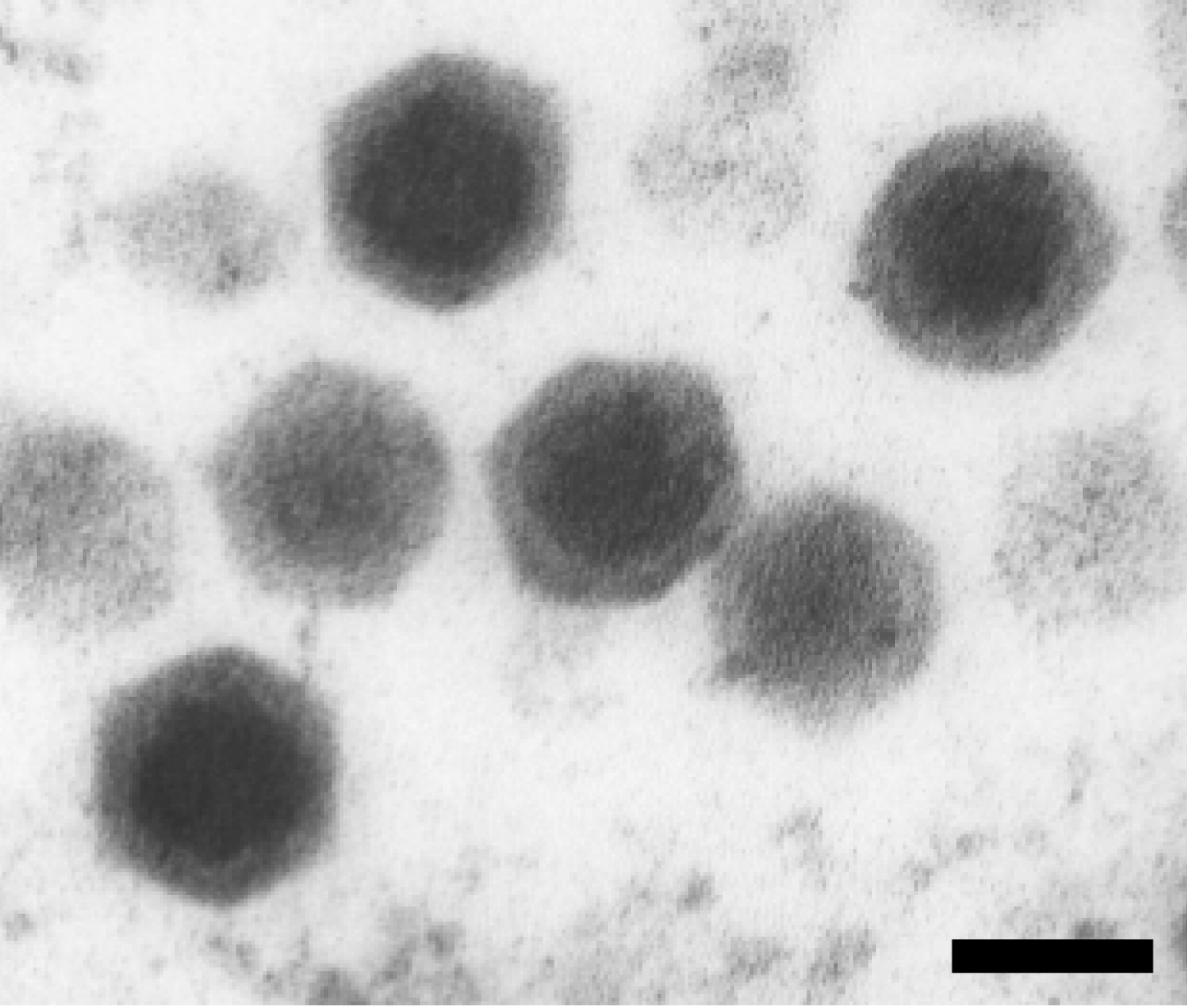
Transmission electron micrograph of IIV22A virions. Bar = 100 nanometers.

The IIV22 and IIV25 isolates originated from a single blackfly larva (*Simulium* spp., order Diptera) collected in 1980 in the Ystwyth river, near Aberystwyth in Wales [11]. They were subsequently propagated in *Aedes* cells in culture, and the virions were purified and plaque assayed using *Spodoptera frugiperda* cells [7]. Large quantities of virions can also be produced using a secondary host, third instar larvae of *Galleria melonella* (order Lepidoptera) [12]. Here, we describe the genome of a variant of IIV22, named IIV22A (Figure 1), which we isolated recently from the same sample. Its analysis revealed that the nucleic acid sequences of the IIV22 and IIV22A genomes are 95 to 100% identical over 98% of their length. Sequence comparisons also showed that the two variants differ by the presence of inverted regions, inteins in certain genes, and transposons, as well as by different numbers of members in several gene families. Altogether, our results suggest that the best criteria to differentiate close IIV species are the divergence rate of their nucleic acid sequences, and differences in the location of certain genes. Indeed, such variations occur between the IIV22 variants.

## Genome Sequencing and annotation

The procedures used for IIV22A and described below are obviously similar to those used for the genome sequencing and annotation of IIV22 and IIV25 [8,9].

### Genome project history

In 2009, the scientific committee of GENOSCOPE selected the IIV31 genome for sequencing. The complete genome sequence and annotation are now available in the EMBL database (HF920634). A summary of the project results are shown in Table 1.

**Table 1:** Genome sequencing project information

**MIGS ID**	**Property**	**Term**
MIGS-31	Finishing quality	Finished (>99%)
	Number of contigs	1
	Assembly size	196455-bp
	Assembly coverage	61 ×
	Total number of read used	31811
MIGS-29	Sequencing plate-form	454
MIGS-30	Assemblers	Newbler version 2.3 Post-release-11.19.2009
	Gene calling method	Annotation protocol [13]
	EMBL ID	HF920634

### Growth conditions and DNA isolation

The Aberystwyth sample containing the Iridovirus type IIV22A [12] was supplied by Professor Trevor Williams (Instituto de Ecologia AC, Xalapa, Mexico) and Professor Primitivo Caballero (Universidad Publica de Navarra, Pamplona, Spain). IIV22A was amplified by infecting third instar larvae of *Spodoptera frugiperda* (order Lepidoptera, family Noctuidae) with a needle. Seven days after infection, larvae were frozen at -80° C. IIV22A virions (Figure1) and genomic DNA (gDNA) were purified as described [14].

### Genome sequencing and assembly

The genome of IIV22A was sequenced using the 454 FLX pyrosequencing platform (Roche/454, Branford, CT, USA). Library construction, and sequencing were performed as previously described [13]. Assembly metrics are described in Table 2. The assembled contig representing the entire IIV22A genome sequence was confirmed by comparing five predicted restriction fragment profiles from the genome, for *Bam*HI, *Eco*RI, *Hin*dIII, *Pst*I and *Sal*I, with the matching fragment profiles produced by actual restriction digestions of the IIV22A genome [10]

**Table 2:** Genome statistics of IIV22A

**Attribute**	**Value**	**% of total^a^**
Genome size (bp)	196,455	100.00
DNA G+C content (bp)	55,204	28.01
DNA coding region (bp)	171,852	87.5
Fossil genes	4	100
Total genes (putatively functional)	174	100
Protein coding genes with function prediction	66	37.9
Protein coding genes with orthologs in databases	171	98.3
Family of gene paralogs	2	-
Genes in families of paralogous genes	24	13.7
Non coding regions over 200 bp in length	6,455 (11 segments)	5.4

^a^The total is based on either the size of the genome in base pairs or the total number of protein coding genes in the annotated genome, where applicable.

### Genome annotation

Genes were identified using the Broad Institute Automated Phage Annotation Protocol as described previously [13,15]. Briefly, evidence based and *ab initio* gene prediction algorithms were used to identify putative genes, followed by construction of a consensus gene model using a rules-based evidence approach. Gene models where manually checked for errors such as in-frame stops, very short peptides, splits, and merges. Additional gene prediction analysis and functional annotation were performed as previously described [16].

## Genome properties

### Genome organization

**Figure 2:**
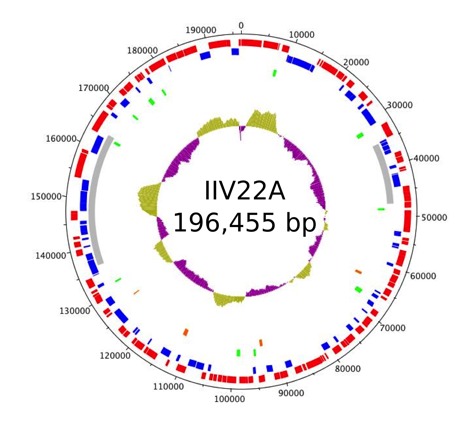
Graphical circular map of the 196,455 bp IIV22A genome. The outer scale is numbered clockwise in bp. Circles 1 and 2 (from outside to inside) denote the coding DNA sequences (CDSs). Forward strands are in red and reverse strands in blue. The grey box in circle 3 represents the two regions that are found in inverse orientation in IIV9. Green boxes in circle 4 represents ORF-free region with a size over 200 bp. The box in orange in circle 5 represents a fossil gene. Circle 6 represents the local variations of G+C content along the genome sequence.

General features of the IIV22A genome sequence Supplementary Table 3 include a nucleotide composition of 28.01% G+C (Figure 2), a total of 174 predicted protein coding genes (CDS). No tRNA gene was found. Of the 174 CDSs, 110 CDS were in forward orientation, 64 in reverse orientation, and no CDS overlapped. Sixty-six CDS (40.1%) have been annotated with functional product predictions.

Pairwise alignment using BLASTn of nucleotide sequences of the IIV22A and IIV22 genomes revealed that they are 95 to 100% identical over 98% of their length. They also revealed that the region spanning from nucleotides 136200 to 163500 in the IIV22A genome is in an inverted orientation in IIV22 between nucleotides 134500 to 158200. Similarly, the alignment of nucleotide sequences of the IIV22A and IIV9 genomes revealed that the same region was inverted in the IIV9 genomes between nucleotides 69000 to 92000. Moreover, the region spanning from nucleotides 35000 to 47500 in the IIV22A genome was found in an inverted orientation in IIV9 between nucleotides 166400 to 177900. Interestingly, the two regions found inverted between IIV22A and IIV9, and IIV30 and IIV9 [17] are the same. Overall, this indicated that the orientations of these two genomic regions are intra and inter specific polymorphisms that very likely pre-existed within the genomes of their common ancestor and which were conserved during the evolutionary differentiation of these IIV species

Since IIV22 and IIV25 may frequently co-habitate in their infected hosts, we have searched for evidence of lateral genetic transfers between genomes of both IIV species. No evidence of such events was located between the genome of IIV25 and those of IIV22A. To some extent, this was unexpected, especially because the IIV22 and IIV25 populations contained in the Aberystwyth sample were amplified several times in noctuid larvae since being isolated. Indeed, it was previously reported for giant viruses belonging to the Megavirale that their replication machinery is very flexible because it has a propensity to allow events of intermolecular recombination during genome replication [18]. Our results indicate that whether such events occurred between IIV22 and IIV25 genomes, they were not sufficiently persistent to be maintained in both virus populations.

### Gene features

The annotation of the 174 genes is described in Table 3. One-hundred seventy one of the 174 protein coding genes have a related gene in databases, with e-values below 10^-3^. The gene content in IIV22 is very close to that of IIV9. Therefore the predicted functional assignments for the 66 protein coding genes are the same as those described by Wong et al. [7]. Only three IIV22A genes 064L, 094R and 119R, have no ortholog. In spite of their proximity, thirteen genes differentiate IIV22 and IIV22A. IIV22 genes 122L, 115L and 145L are absent in the IIV22A genome, and IIV22A genes 004R, 058R, 064L, 094R, 0103R, 119R, 0128L, 0136L, 150R and 0165L are absent in the IIV22 genome. Obviously, none of these genes are NLCDV core genes [3]. These variations have two origins. The DNA regions containing the IIV22 genes 122L, 115L and 145L and the IIV22A genes 004R, 064L, 094R, 0103R, 119R and 0136L are absent in their variant counterparts. IIV22 regions corresponding to IIV22A genes 058R, 150R and 165L are conserved but contained few stop codon or frame shifts that have disrupted these ORFs.

In regard to repeats, whereas three families of gene paralogs occur in the IIV22 genome, only two were found in IIV22A. The first contains 16 members that are related to CIV genes 006L, 019R, 029R, 146R, 148R, 211L, 212L, 238R, 313L, 388R, 420R and 468L. The second contains 8 members related to CIV261R, 396L and 443R. No member of the *bro*-like gene family was found in the IIV22A genome, whereas 2 are present in that of IIV22 [19]. Four pseudogenes were found in IIV22A. Their status was confirmed by polymerase chain reaction and sequencing, so we annotated these as fossil genes. The first is located between CDS 057R and 058R is a remnant member of first family of gene paralogs related to CIV genes 006L, 019R, 029R, 146R, 148R, 211L, 212L, 238R, 313L, 388R, 420R and 468L. The three other fossils were located, respectively, between CDS 061L and 062L, 087L and 088R, and 119R and 120R.

The cluster of five co-linear genes in IIV3 (028R to 032R), IIV9 (097R to 101R), IIV22 (148R to 152R), and IIV25 (158R to 162R) was found conserved in IIV22A (154R to 158R). However, it was found interrupted, as in IIV30 [17], by a non-coding DNA segment of 1260-bp inserted between CDS 156L and 157R. The non-coding DNA segment of 1260-bp in IIV22A, however, is different from that found at the same position in IIV30 (2690-bp).

Overall, our analysis of the genes present in the IIV22 and IIV22A genomes indicates that about 7.5% of the genes show a presence/absence between both variants that are due to gene insertions or deletions, or to the accumulation of few punctual mutations. They also indicate that the presence or the absence of non-core genes cannot be used as a criterion to differentiate virus species within the Polyiridovirus complex since variant and viral species show similar ranges of differences.

### Mobile DNA elements

The presence of certain mobile genetic elements that occur in some NLCDVs belonging to the families Phycodnaviridae and Mimiviridae [20] was searched for in the IIV22A genome. As already observed in IIV22, IIV25 and IIV30 [8,9,17] no transpovirons and group I introns were found. No intein was found inserted into the ORF 001R of IIV22A. However, one intein was found to be specifically inserted in frame in 097R, as reported [21].

**Figure 3:**
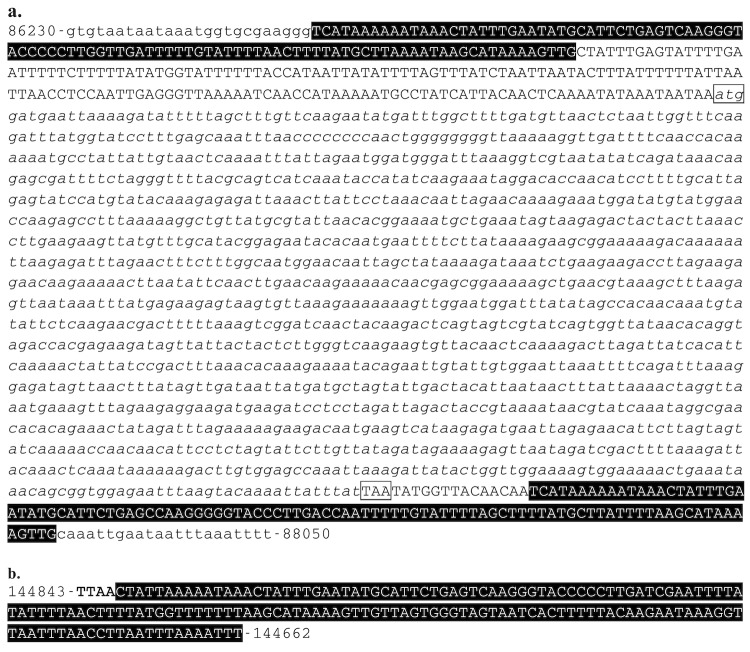
Nucleotide sequence of IIV22-MITE halves found in the IIV22A genome between nucleotides 86230 to 88050 (a) and 144843 to 144662 (b). IIV22-MITE ITR at both ends are highlighted in black and typed in white. In a, the CDS082 is in italics, its start and stop codons are boxed. In b, TTAA at the insertion site is in bold type.

In contrast to IIV9 and IIV22 [7,8], no full-length MITEs or Class II DNA transposon were found in the IIV22A genome. Nevertheless, we have found two halves of the IIV22-MITE in a head to tail orientation flanking at both ends the CDS 082R (Figure 3a, Table 3) that is member of the family of gene paralogs related to CIV genes 006L, 019R, 029R, 146R, 148R, 211L, 212L, 238R, 313L, 388R, 420R and 468L. We also found one solo 5’half of the IIV22-MITE between nucleotides 144843 and 144662 (Figure 3b). Together, this suggests that the IIV22-MITE may serve as a recombination factor in the distribution and the dynamics of members of some families of gene paralogs in these viruses. It also indicates that the IIV22-MITE is not a recent visitor of the IIV22 genomes. Together with the knowledge accumulated with the genome of IIV9, IIV22, IIV25 and IIV30, it appears that the IIV9-MITE and the IIV22-MITE have been respectively acquired by IIV9 and IIV22 since their speciation from the common ancestor of these four iridoviruses.

## Conclusions

IIV22A is the seventh genome of an IIV to be sequenced and reported. Many of the CDSs identified display high conservation with their counterparts in other IIVs, insect and bacterial genomes. Further sequencing of related strains will no doubt reveal more about the genetic and functional diversity of these viruses.

IIV22A is a variant of IIV22 and was isolated from the same original sample. Analysis of its genome enabled us to determine how this isolate differed from IIV22. To summarize, these differences are that IIV22A contains more CDS than IIV22 (174 versus 167), the features of the families of gene paralogs are different in terms of the number of families as well as in the number of members per family. IIV22A also has two long genomic regions that are in an inverted orientation, has no intein inserted in its CDS 001R, and the IIV22A-MITE has a different profile from that of IIV22.

We noted previously that the presence of a eukaryotic Class 2 DNA transposon in the IIV9 and IIV22 indicated that iridoviruses could be vectors for horizontal transfer of transposable elements between host species. Interestingly, the genome analyses of the IIV22 and IIV22A genome suggest that MITEs could be recombination factors controlling the distribution and dynamics of some families of gene paralogs in these viruses.
